# Computational Language Modeling and the Promise of In Silico Experimentation

**DOI:** 10.1162/nol_a_00101

**Published:** 2024-04-01

**Authors:** Shailee Jain, Vy A. Vo, Leila Wehbe, Alexander G. Huth

**Affiliations:** Department of Computer Science, University of Texas at Austin, Austin, TX, USA; Department of Neuroscience, University of Texas at Austin, Austin, TX, USA; Brain-Inspired Computing Lab, Intel Labs, Hillsboro, OR, USA; Machine Learning Department, Carnegie Mellon University, Pittsburgh, PA, USA; Neuroscience Institute, Carnegie Mellon University, Pittsburgh, PA, USA

**Keywords:** computational neuroscience, deep learning, encoding models, experimental design, natural language processing, naturalistic stimuli

## Abstract

Language neuroscience currently relies on two major experimental paradigms: controlled experiments using carefully hand-designed stimuli, and natural stimulus experiments. These approaches have complementary advantages which allow them to address distinct aspects of the neurobiology of language, but each approach also comes with drawbacks. Here we discuss a third paradigm—in silico experimentation using deep learning-based encoding models—that has been enabled by recent advances in cognitive computational neuroscience. This paradigm promises to combine the interpretability of controlled experiments with the generalizability and broad scope of natural stimulus experiments. We show four examples of simulating language neuroscience experiments in silico and then discuss both the advantages and caveats of this approach.

## INTRODUCTION

One major goal of language neuroscience is to characterize the function of different brain regions and networks that are engaged in language processing. A large body of work has investigated different aspects of language processing—such as semantic knowledge representation (Binder et al., 2009; Huth et al., 2016; Mitchell et al., 2008), syntactic processing (Friederici et al., 2000), and phonological mapping (Chang et al., 2010)—and characterized the properties of the language network like the processing timescale (Lerner et al., 2011), convergence with different sensory systems (Popham et al., 2021), role in bilingual representations (Chan et al., 2008), and more. To study these questions, language neuroscientists have developed a suite of experimental designs, ranging from highly specific controlled experiments to natural stimulus experiments and, more recently, deep learning-based approaches for computational modeling.

Each experimental design can be thought of as an investigative tool for understanding the brain’s response *R*_*v*_ = *f*_*v*_(*S*), where *f*_*v*_ is the function that some brain element *v* (e.g., a single neuron, voxel, brain area, or magnetoencephalography [MEG] sensor) computes over a given language stimulus *S* to produce responses *R*_*v*_. Some experimental designs—like contrast-based studies—aim to directly compare certain aspect of *f*_*v*_, such as the response to different word categories. Others—like experiments with complex stimuli that are paired with encoding models—approximate *f*_*v*_ using computational tools, and this allows for the prediction of activity related to new stimuli. In this paper we describe an alternative to existing paradigms: in silico controlled experimentation using computational models of naturalistic language processing. This hybrid approach combines the strengths of controlled and naturalistic paradigms to achieve high ecological generalizability, high experimental efficiency and reusability, high interpretability, and sensitivity to individual participant effects.

We first compare and contrast experimental designs based on their effectiveness and efficiency for revealing *f*_*v*_. Then we introduce the in silico experimentation paradigm with deep learning models. We discuss four different neuroimaging studies that use this paradigm to investigate different linguistic phenomena in the brain. And finally, we discuss the potential of this approach to alleviate the problems of reproducibility in language neuroscience, as well as caveats and pitfalls of in silico experimentation.

## EXPERIMENTAL DESIGNS IN LANGUAGE NEUROSCIENCE

### Controlled Experimental Design: Contrast-Based Studies

Language is a rich and complex modality that humans are uniquely specialized to process. Given this complexity, neuroscientists have traditionally broken language down into specific processes and properties and then designed controlled experiments to test each separately (Binder et al., 2009; Friederici et al., 2000). Consider the example of investigating which areas of the brain are responsible for encoding specific types of semantic categories like “actions” (Kable et al., 2002; Noppeney et al., 2005; Wallentin et al., 2005). A simple and effective approach is to collect and compare brain responses to action words and pair them with minimally different words, perhaps similar length and frequency “object” words. If some brain element *v* responds more to stimuli containing the property being tested than the control stimuli—that is, *f*_*v*_(“action” words) > *f*_*v*_(“object” words)—the experimenter concludes that *v* is involved in processing action words. Similarly, the N400 effect (Kutas & Hillyard, 1984) is assessed by testing whether an elements’ *f*_*v*_ reflects surprise with respect to some context. If *f*_*v*_(expected word|context) < *f*_*v*_(unexpected word|context), it would suggest that the brain element is capturing word surprisal.

In order for a contrast-based study to be interpretable, it is vital to remove any confounds that could corrupt observed responses and lead to false positives. Binder et al. (2009) characterize three types of confounds: the main and control conditions could differ in low-level processing demands (phonological/orthographic); the main and control conditions could differ in working memory demands, attention demands, and so forth; and, in passive tasks, the participants might engage in different mental imagery or task-related thoughts in the two conditions. If such confounds are controlled effectively, one can assume that the observed brain response will be identical in all respects unless *v* specifically captures the property being studied. For example, if the action and object words are matched on all other properties, *f*_*v*_(“action” words) and *f*_*v*_(“object” words) will only differ if *v* selectively encodes action or object concepts. Consequently, the contrast-based paradigm has high interpretability, as any variations in observed response can be attributed to the hypothesis. This clear and direct relationship between hypothesis and result ensures that the experiment has scientific value even when a hypothesis or theory is incorrect. The controlled experimental design has thus been fundamental in revealing many important aspects of brain function, such as the specialization of parts of temporal cortex for speech processing (reviewed in S. K. Scott, 2019) and distinct neural systems for concrete versus abstract concepts (Binder et al., 2005; Binder et al., 2009).

While this paradigm has been hugely influential and effective in language neuroscience, it is not without flaws. Perhaps the biggest drawback of most contrast-based designs is the lack of ecological generalizability (Hamilton & Huth, 2018; Matusz et al., 2019). To avoid confounds, controlled experiments often employ the simplest linguistic constructions required to demonstrate an effect, such as single words in the action versus object contrast. While we are fully capable of identifying action words in isolation, it is not necessary that the brain employs the same networks to understand such words in real-world settings (Matusz et al., 2019), for example, as used in a conversation or a story. In contrast to such studies, those using naturalistic stimuli have found more engagement and activation in higher order cortical regions, likely due to the incorporation of long-range structure (Deniz et al., 2021; Lerner et al., 2011). Furthermore, due to practical limitations, controlled studies typically use small stimulus sets that span a limited domain. For example, neuroimaging studies of the action contrast often use fewer than 100 words in each condition. This raises the probability that there is something peculiar or nonrepresentative about the experimental stimuli, making it more difficult to reproduce the effect or establish generalizability to a broader stimulus domain (Yarkoni, 2022). Small stimulus sets can also artificially inflate the observed statistical significance (Westfall et al., 2017).

While controlled studies offer a very clear and direct relationship between the hypothesis and experimental result, their value depends entirely on the quality of the hypothesis. In many cases, narrowing the experimental hypothesis to focus on contrasts of a particular stimulus property may be misleading, and may fail to account for interactions between several other stimulus properties. For example, standard statistical models for assessing the “action” contrast assume that brain response is identically distributed for any subcategorization of this semantic concept. However, studies such as Hauk et al. (2004) have found that different regions across cortex selectively encode hand-related, foot-related, or mouth-related actions. This type of subcategory specificity decreases the statistical power of the overall action contrast, thereby increasing the probability of false negatives. Worse, if the overall action contrast has unevenly sampled these subcategories, the statistical power to detect action selectivity will vary in an unexpected and unknown fashion between brain areas. This issue can occur in any contrast-based experiment and is difficult or even impossible to detect by the experimenter. One potential solution would be to combine data across different contrast-based experiments, which could reveal interactions between effects. However, separate controlled experiments often do not share analysis methods, stimulus sets, or subjects, making it difficult to combine data or compare effect sizes across experiments. Lastly, for each language property that one wishes to investigate using a controlled experiment, one needs to design specific controls and repeatedly measure *R*_*v*_. This results in limited reusability of experimental data, slowing down the process of scientific discovery.

### Naturalistic Stimuli

To combat the lack of stimulus generalization and limited reusability, there has been a rising trend toward naturalistic experimental paradigms (Brennan et al., 2012; Hamilton & Huth, 2018; Hasson et al., 2008; Lerner et al., 2011; Regev et al., 2013; Shain et al., 2020). With the development of better neuroimaging/recording technology, we now have access to high quality brain recordings of humans while they perceive engaging, ecologically valid stimuli like podcasts (Huth et al., 2016; Lerner et al., 2011; J. Li et al., 2022; Nastase et al., 2021; S. Wang et al., 2022), fictional books (Bhattasali et al., 2020; Wehbe, Murphy, et al., 2014), and movies (J. Chen et al., 2017)—all examples of stimuli humans encounter or seek out in their everyday lives. Recent work has further developed this naturalistic paradigm to incorporate communication and social processing, beyond passive perception (Bevilacqua et al., 2019; Redcay & Moraczewski, 2020). Naturalistic stimulus data sets are easier to construct and often larger than controlled stimuli. For example, J. Chen et al. (2017) publicly released a data set collected on a 50 min movie, Wehbe, Murphy, et al. (2014) released data collected on an entire chapter from the Harry Potter books, comprising more than 5,000 words, and LeBel et al. (2022) released data collected on over 5 hr of English podcasts per participant. These stimuli also provide a diverse test bed of linguistic phenomena—from a broad array of semantic concepts to rich temporal structure capturing discourse-level information. Furthermore, they do not directly constrain the hypotheses the experimenter can test and thus facilitate high reusability of the data. However, this also means that natural stimulus data have low statistical power with respect to any specific hypothesis, and it is necessary to carefully design analyses to control for confounding effects. This makes interpretation of the observed effects much more challenging than contrast-based experiments.

### Naturalistic Experimental Design: Controlled Manipulations of Naturalistic Stimuli

To reap the benefits of both interpretable controlled experiments and generalizable naturalistic stimuli, some studies have deployed a hybrid experimental design (Chien & Honey, 2020; Deniz et al., 2019; Lerner et al., 2011; Overath et al., 2015; Yeshurun et al., 2017). Here, natural stimuli are manipulated to change or remove some specific language cue or property (e.g., scrambling the words in a story) and the sensitivity of different brain regions to this manipulation is measured, for example, *f*_*v*_(intact story) vs. *f*_*v*_(scrambled story). This can reveal properties across the brain like the timescale of information represented (Lerner et al., 2011, 2014) or specificity to the type of naturalistic stimulus, such as human speech (Overath et al., 2015). This experimental design accounts for ecological validity by restricting analyses to brain regions that robustly respond to the naturalistic stimuli. Furthermore, it has the same advantage of controlled experiments when it comes to interpretation: Assuming effective control of confounds, any observed change in brain activity is likely to be an effect of the stimulus manipulation. However, this approach also has disadvantages: The manipulated stimuli are often unnatural (like reversed or scrambled speech) and restrict the types of interactions the experimenter can observe. For example, the scrambled story experiment assumes that all regions processing short timescale information will behave identically. The manipulated stimuli also limit the reusability of the experiment, meaning that a new experiment needs to be designed for each effect of interest.

### Naturalistic Experimental Design: Predictive Computational Modeling

Encoding models are an alternative computational approach for leveraging naturalistic experimental data (Bhattasali et al., 2019; Caucheteux & King, 2022; Goldstein et al., 2021; Huth et al., 2016; Jain et al., 2020; Jain & Huth, 2018, p. 20; Schrimpf et al., 2021; Wehbe, Vaswani, et al., 2014). These predictive models learn to simulate elicited brain responses *R*_*v*_ = *f*_*v*_(*S*) to natural language stimuli *S* by building a computational approximation to the function *f*_*v*_ for each brain element *v*, typically in every participant individually. Here, *R* can be captured by any neuroimaging or neural recording technique. Given limitations on data set sizes, the search for *f*_*v*_ is typically constrained to *linearized encoding models*, *g*_*v*_(*Ls*(*S*)) (M. C.-K. Wu et al., 2006), where *g*_*v*_ is a linear combination of features extracted from the stimulus by a function *Ls*. While *g*_*v*_ is termed a *linear model*, of particular interest is the *linearizing transform Ls*. Contrast-based experimental designs test a hypothesis by comparing responses elicited by different conditions. Each condition is composed of stimuli that share some features (e.g., all words that describe actions). Encoding models can test the same hypothesis by incorporating these features into *Ls*. For example, for every word in the natural stimulus, one could create an indicator feature *I*_action_ that is 1 if the word describes an action and 0 otherwise. Feature spaces consisting of 1s and 0s are equivalent to a contrast-based experimental design, assuming other confounds have been eliminated.

Encoding models can also adopt much more complex and high-dimensional functions for *Ls*. This makes it possible to account for multiple, interacting stimulus properties that may affect the response *R*_*v*_. For example, *Ls* could indicate multiple levels of semantic categories. In the example of action and object words, the feature space could indicate that hand-related, foot-related, and mouth-related words were all types of actions, and distinguish all action words from multiple subcategories of objects. One recent example of such a high-dimensional feature space that captures semantic similarity (Mikolov et al., 2013; Pennington et al., 2014) is *word embeddings*, which have been used to characterize semantic language representations across the human brain (de Heer et al., 2017; Huth et al., 2016; Wehbe, Murphy, et al., 2014; Wehbe, Vaswani, et al., 2014). With a suitably rich linearizing transform *Ls*, this approach vastly expands the set of hypotheses that can be reasonably explored with a limited data set. The expandable feature space also allows encoding models great flexibility to test additional hypotheses without collecting new data, leading to high reusability. Estimating the brain response as a function of the nonlinear feature space is made possible by collecting large data sets that are partitioned into a portion for training (estimating) the model and a portion for testing the model on unseen data. Typically, regularized linear regression is used to estimate the linear relationship *g*_*v*_ based on the feature space *Ls*. This is used to predict new responses

$$\hat{R_{v}}=g_{v}\left(Ls\left(S\right)\right)$$

to unseen stimuli. Finally, the model is evaluated by measuring how well it predicts brain responses, *ρ*(*f*_*v*_(*S*_new_), *g*_*v*_(*Ls*(*S*_new_))). Thus, unlike other approaches, encoding models explicitly measure generalizability by testing on new, naturalistic stimuli. In contrast-based designs, a generalization test is usually achieved through replication with an independent data set, often from a different lab where protocols and analysis details may differ. With encoding models, the same experimenter usually runs their own generalization test and directly estimates how much of the neural response *R*_*v*_ is explained by the model, holding all other variables constant. Encoding models can also be used to investigate if the same brain region under different tasks have the same tuning. For example, Deniz et al. (2019) show that semantic tuning is preserved between reading and listening, while Çukur et al. (2013) show that the tuning of different regions in visual cortex when attending to a given category is biased toward the attended category. Encoding models can also be used to compare tuning of two different regions (Toneva et al., 2022).

### Artificial Neural Networks as a Rich Source of Linguistic Features

The most important choice that an experimenter makes when using encoding models is that of the linearizing transform. To find useful linearizing transforms, neuroscience has mostly followed advances in computational linguistics or natural language processing (NLP) where, in recent years, deep learning (DL) models trained using self-supervision have seen great success. One such cornerstone model is the *neural language model*—a self-supervised artificial neural network (ANN) that learns to predict the next word in a sequence, *w*_*t*+1_, from the context provided by previous words (*w*_1_, *w*_2_ … *w*_*t*_). Several recent studies have shown that representations derived from LMs capture many linguistic properties of the preceding sequence (*w*_1_, *w*_2_ … *w*_*t*_) like dependency parse structure, semantic roles, and sentiment (see Mahowald et al., 2020, for a review; Clark et al., 2019; Conneau et al., 2018; Gulordava et al., 2018; Haber & Poesio, 2021; Hewitt & Liang, 2019; Hewitt & Manning, 2019; Lakretz et al., 2019; B. Z. Li et al., 2021; Linzen & Leonard, 2018; Marvin & Linzen, 2018; Prasad et al., 2019; Tenney et al., 2018; Tenney et al., 2019). While this by no means is a complete representation of phrase meaning (Bender & Koller, 2020), using a language model as a linearizing transform has been shown to effectively predict natural language responses in both the cortex and cerebellum, with different neuroimaging techniques and stimulus presentation modalities (Abnar et al., 2019; Anderson et al., 2021; Caucheteux & King, 2022; Goldstein et al., 2021; Jain et al., 2020; Jain & Huth, 2018; Kumar et al., 2022; LeBel et al., 2021; Reddy & Wehbe, 2020; Schrimpf et al., 2021; Toneva et al., 2020; Toneva & Wehbe, 2019; S. Wang et al., 2020; Wehbe, Murphy, et al., 2014; Wehbe, Vaswani, et al., 2014). Moreover, these models easily outperform earlier word embedding encoding models that use one static feature vector for each word in the stimulus and thus ignore the effects of context (Antonello et al., 2021; Caucheteux & King, 2022; Jain & Huth, 2018). Deep LMs have also been used to investigate the mapping between ANN layer depth and hierarchical language processing (Jain & Huth, 2018). Along similar lines and at a lower level, supervised and self-supervised models of speech acoustics have been used to develop the best current models of auditory processing in human cortex to date (Kell et al., 2018; Y. Li et al., 2022; Millet et al., 2022; Millet & King, 2021; Vaidya et al., 2022).

The unprecedented success of DL-based approaches over earlier encoding models can likely be attributed to several important factors. First, features extracted from the DL-based models have the ability to represent many different types of linguistic information, as discussed above. Second, DL-based models serially process words from a language stimulus to generate incremental features. This mimics causal processing in humans and thus offers an important advantage over static representations like word embeddings, which cannot encode contextual properties. Third, recent work has shown that these models often recapitulate human errors and judgments, such as effectively predicting behavioral data of human reading times (Aurnhammer & Frank, 2018; Futrell et al., 2019; Goodkind & Bicknell, 2018; Merkx & Frank, 2021; Wilcox et al., 2021). This again suggests some isomorphism between human language processing and DL-based models. The next word prediction objective also enables language models to perform well on psycholinguistic diagnostics like the cloze task, although there is substantial room for improvement (Ettinger, 2020; Pandia & Ettinger, 2021). Finally, self-supervised ANNs, that is, networks that predict the next word or speech frame, transfer well to downstream language tasks like question answering and coreference resolution, and to speech tasks like speaker verification and translation across languages (Z. Chen et al., 2022; A. Wu et al., 2020). This suggests that the self-supervised networks are learning representations of language that are useful for many tasks that humans may encounter.

These factors have contributed to the increasing popularity of DL-based encoding models as an investigative tool of brain function. This approach has revealed aspects of how the brain represents compositional meaning (Toneva et al., 2020), provided fine-grained estimates of processing timescales across cortex (Jain et al., 2020), and uncovered new evidence for the cerebellum’s role in language understanding (LeBel et al., 2021).

Yet despite these successes, DL-based encoding models are hard to interpret. The representations produced by language models are entirely learned by black-box neural networks, and thus cannot be understood with the same ease as the indicator features described in the Naturalistic Experimental Design: Predictive Computational Modeling section above. While the representations themselves are opaque, one potential avenue is to interpret the success of a DL-based model at predicting some brain area as suggesting a commonality between that brain area and the objective that model was trained for (e.g., word identification [Kell et al., 2018] or 3D vision tasks [A. Wang et al., 2019]). However, the fact that similar representations can be derived from DL-based models that are trained for different objectives puts this type of interpretation on shaky ground (Antonello & Huth, 2024; Guest & Martin, 2023). These difficulties have left the field at something of an impasse: We know that DL-based models are extremely effective at predicting brain responses, but we are unsure why and unsure what these models can tell us about how the brain processes language.

### Pièce De Résistance: In Silico Experimentation With DL-Based Encoding Models

Controlled experiments and encoding models using naturalistic stimuli both have distinct advantages and disadvantages. However, it may be possible to combine these paradigms in a way that avoids the disadvantages and retains the advantages. To this end, we present an experimental design that combines these two paradigms: in silico controlled experimentation using encoding models. This paradigm first trains encoding models on an ecologically valid, highly generalizable naturalistic experiment. Then, it uses the encoding models to simulate brain activity to controlled stimulus variations or contrasts. Notably, this does not require additional data to be collected for every condition.

The first use of in silico experimentation is to test if effects discovered in controlled, non-ecologically valid setups generalize to naturalistic stimuli. This experimental design also facilitates quick and efficient hypothesis testing. Experimenters can prototype new controlled experiments and narrow down the desired contrasts or stimuli without having to repeatedly measure in vivo. While this is a complement to and not a substitute for in vivo experiments that should follow the prototyping phase, in silico experimentation can greatly reduce the cost of generalizability and hypothesis testing, and accelerate scientific discovery.

**Figure 1.**
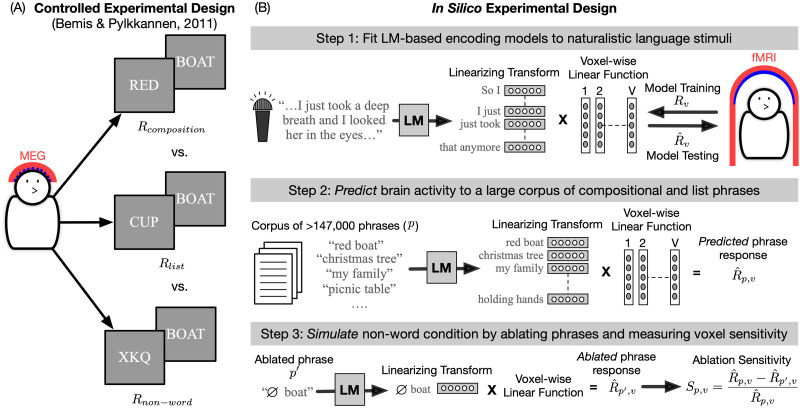
Example of an in silico adaptation of a controlled experiment. (A) The original MEG study investigated composition over two-word phrases (Bemis & Pylkkänen, 2011). This was done by presenting three different types of phrases to participants to solve a picture matching task. By contrasting the elicited brain responses in the composition condition with the responses in the list and non-word conditions, the authors could infer which brain regions are engaged in compositional processing of two-word phrases. (B) This experimental paradigm can be conceptually simulated with LM-based fMRI encoding models of naturalistic stimuli. The composition and list conditions can be tested by using the learned encoding model to predict each voxel’s response to a large, diverse corpus of phrases. The non-word condition can be simulated by replacing the first word in a phrase with a non-word, extracting new *ablated* features of the phrase from the LM and using the encoding model to predict the brain’s response to the ablated phrase. If a voxel’s response is highly sensitive to the removal of the first word, it would suggest that the voxel combines information over both words to arrive at meaning. This provides a data-efficient way to test for compositional processing across diverse types of phrase constructions. fMRI = functional magnetic resonance imaging; LM = language model; MEG = magnetoencephalography.

In Figure 1, we present a controlled experimental design with its in silico counterpart. Figure 1A shows an experimental paradigm that was designed to understand linguistic composition of two-word phrases (Bemis & Pylkkänen, 2011). Participants were presented with phrases in which meaning can be composed across constituent words and contrasting conditions where it cannot (word list and non-word). This experiment can be conceptually simulated in silico, as shown in Figure 1B (Jain & Huth, 2023). Instead of collecting separate neuroimaging data for each type of phrase construction, the in silico experiment was done with DL-based encoding models trained on two-word sequences. The learned models were first used to predict brain responses to a large, diverse corpus of phrases that contained both noun–noun and adjective–noun constructs among others. Next, the non-word condition was simulated by replacing the first word in the phrase with a non-word, extracting a new *ablated* feature, and finally predicting each functional magnetic resonance imaging (fMRI) voxel’s response to the ablated phrase. Assuming that the DL-based encoding model captures compositional effects, this in silico experiment can ameliorate the disadvantages of both controlled and encoding model-based experimental designs. First, since simulating responses is trivial in both time and cost, the simulated experiment can use thousands or even millions of two-word phrases instead of the hundreds that can be tested in vivo. This ameliorates problems that arise with limited stimulus sets that may fail to account for key properties or generalize to naturalistic contexts. Second, by simulating and then comparing responses under conditions that are derived from linguistic theory (composition vs. single word, or word list), this in silico experiment provides results that are easily and explicitly interpretable, unlike encoding models with natural stimuli. However, one major concern raised by this approach is whether the encoding model can capture how the brain responds to the language properties of interest. To address this it is important to verify both that the encoding model is highly effective at predicting brain activity, and that it is sufficiently complex to capture the desired property.

Similar in silico experimentation has recently become popular in vision neuroscience. There, DL-based encoding models of the visual system are first trained on ethologically valid tasks like object recognition. Then they are probed to understand brain function (Yamins & DiCarlo, 2016). For example, Bashivan et al. (2019) used DL-models to synthesize images that maximally drive neural responses. This provided a noninvasive in silico technique to control and manipulate internal brain states. Similarly, Ratan Murty et al. (2021) synthesized images from end-to-end DL models trained on brain data to provide stronger evidence for the categorical selectivity of different brain regions. In silico experimentation with explicit computational models has also been used in studies of the medial temporal lobe. In Nayebi et al. (2021), computational models of the medial entorhinal cortex were used to investigate the functional specialization of heterogeneous neurons that do not have stereotypical response profiles. By doing ablation in silico, they found that these types of cells are equally important for downstream processing as are grid- and border-cells. Each of these studies first relied on the encoding model’s ability to generalize to new stimuli. This was an indication that the features learned by the DL-based models encoded similar information to the brain regions that they predicted well. Second, these studies leveraged the predictive ability of encoding models to simulate brain activity in new, controlled conditions as a lens into brain function. This enabled the researchers to explore aspects of brain function that would otherwise be highly data intensive or impossible to do.

In language, in silico experimentation is a promising area that is under development, bolstered by the successes in vision neuroscience and growing efforts to understand artificial language systems. One of its earliest uses is the BOLDpredictions simulation engine (Wehbe et al., 2018; Wehbe et al., 2021), an online tool that allows the user to simulate language experiments that contrast two conditions, each defined by a list of isolated words. BOLDpredictions relies on an encoding model from a natural listening experiment that predicts brain activity as a function of individual word embeddings (Huth et al., 2016). In the following sections, we review in silico adaptations of four different language experiments based on four separate data sets. Each of these in silico experiments uses a single naturalistic experiment to train the encoding models, illustrating how a single data set and experimental approach can provide a flexible test bed for many different hypotheses about natural language. The first experiment uses the BOLDpredictions engine to simulate a semantic contrast comparing concrete and abstract words (Binder et al., 2005), testing its generalizability to naturalistic settings. The next experiment focuses on a contrast-based study of composition in two-word phrases (Bemis & Pylkkänen, 2011), testing generalizability over a broader, more diverse stimulus set. The third experiment adopts contrasts from a group-level study investigating the temporal hierarchy for language processing across cortex by manipulating naturalistic stimuli (Lerner et al., 2011). This simulation checks if effects persist at the individual-level and demonstrates how a successful replication can be used to validate computational model constructs themselves. Finally, the last experiment conceptually replicates a study on forgetting behavior in the cortex that also uses controlled manipulations of naturalistic stimuli (Chien & Honey, 2020). This simulation demonstrates the possibility of misinterpretation with the in silico approach, arising from fundamental computational differences between neural language models and the human brain.

In the experimental simulations described below, voxelwise encoding models were fit to fMRI data collected from a naturalistic speech perception experiment. Participants listened to natural, narrative stories from *The Moth Radio Hour* (Allison, 2009–) while their whole-brain BOLD responses were recorded (*N* = 8 for study 1; *N* = 7 for studies 2 and 3). In each study, encoding models were fit for each voxel in each subject individually using ridge regression. The learned models were then tested on one held-out story that was not used for model estimation, and encoding performance was measured as the linear correlation between predicted and true BOLD responses. Statistical significance of the encoding performance was measured using temporal blockwise permutation tests (*p* < 0.001, false discovery rate (FDR) corrected; Benjamini & Hochberg, 1995). Finally, in silico analyses were conducted on voxels that were significantly predicted by the encoding model, broadly covering the temporal, parietal, and frontal lobes.

#### Semantic contrasts: Wehbe et al. (2018)

Binder et al. (2005) investigated the brain regions responsive to abstract and concrete concepts. Subjects read individual stimulus strings and pressed one of two buttons to indicate whether each one was a word or a non-word. The study reported that concrete words activated bilateral language regions such as the angular gyrus more than abstract words, and abstract words activated left inferior frontal regions more than concrete words. In total, the authors found 15 cluster peaks.

**Figure 2.**
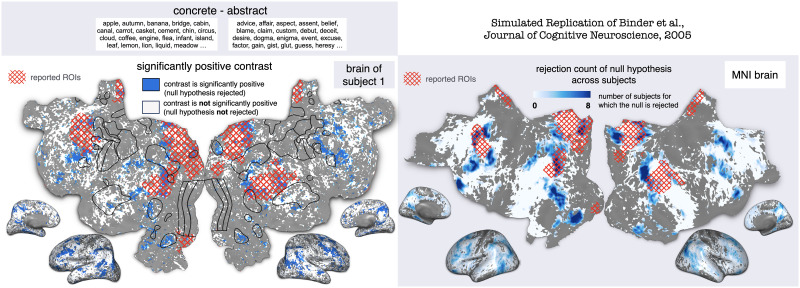
Generalizability test using BOLDpredictions of the concrete vs. abstract contrast of Binder et al. (2005). The authors compared fMRI activity when subjects processed concrete and abstract words. Wehbe et al. (2018) used the published stimulus to simulate the contrast for each subject and run a permutation test. After MNI space transformation, the number of subjects for which the null hypothesis was rejected is computed at each voxel. The simulated statistical maps are shown on flattened maps and inflated 3D hemispheres. Results for subject 1 are shown in subject 1’s native cortical space. Results for the average of eight subjects are shown in the MNI space. Published ROIs are estimated as 10 mm radius spheres, shown in red hatch on the flatmaps (distortion due to the flattening process). A comparison of the overlap of the reported ROIs and the statistical maps reveals that Wehbe et al. (2018) achieve a relatively high overlap for specific ROIs (in the angular gyri, in the posterior cingulate gyri, the right precuneus, and the middle temporal gyri) and not for others. Therefore, BOLDpredictions predicts that the contrast from Binder et al. (2005) generalizes to naturalistic conditions, to a certain extent. MNI = Montreal Neurological Institute; ROIs = regions of interest.

Wehbe et al. (2018) evaluated the reproducibility of these results using an encoding model trained on naturalistic stimuli. They simulated a contrast between the lists of concrete words and abstract words that were kindly shared by Binder et al. (2005). Figure 2 shows the significance map for subject 1 and the group-averaged significance map showing the number of subjects for which the null hypothesis is rejected. The reported regions of interest (ROIs) are shown as an overlay on the flattened cortical maps. Each ROI is originally reported as a single coordinate in brain space and is estimated to have a radius of 10 mm. For every one of the eight subjects, many voxels were significantly more activated for concrete words over abstract words (with *p* < 0.05, FDR corrected permutation test over the words in each condition), specifically in areas bordering the visual cortex and parts of the inferior frontal gyrus. Some reported ROIs had a high overlap with the significance map (specifically in the angular gyri, in the posterior cingulate gyri, the right precuneus, and the middle temporal gyri). The significant effect in those ROIs can be considered to be replicated by BOLDpredictions. However, the reported ROIs and the significance map did not always agree, with the effect in some regions being reported only by Binder et al. (2005) or only by Wehbe et al. (2018).

There are many possible reasons for non-generalizability of individual reported ROIs, including the stochasticity of brain activity, variations in experimental paradigms and analysis techniques, and lack of reproducibility. The authors of BOLDpredictions (Wehbe et al., 2018; Wehbe et al., 2021) note that any scientific finding needs to be reproduced in a series of experiments that would create a body of evidence toward this finding, and the in silico experimentation using BOLDpredictions is one additional piece of evidence. The authors also note that expanding the engine to different data sets, models, and so forth will establish the robustness of the in silico effects and help determine if the original contrast-based experiment lacks reproducibility (Wehbe et al., 2018).

#### Semantic composition contrasts: Jain and Huth (2023)

In the second in silico experiment, Jain and Huth (2023) simulated and expanded on studies of combinatorial processing in 2-word phrases across cortex, first described in Bemis and Pylkkänen (2011). The original controlled experiment consisted of participants reading two word adjective–noun phrases (“red boat”) and doing a picture matching task while brain responses were recorded using MEG. To contrast this compositional condition, a list control was introduced wherein participants were presented with two-word noun–noun phrases (“cup boat”) along with a non-word control consisting of a non-word and a word (“xkq boat”). Note that participants were instructed to avoid composing meaning in the word list, but no explicit control was introduced. To isolate regions involved in two-word composition, the study contrasted the adjective–noun condition with the controls. The experimenters tested 25 base nouns, six color adjectives, and six non-words. Overall, they found that areas in ventral medial prefrontal cortex (vmPFC) and left anterior temporal lobe both selectively responded to the composition condition.

Jain and Huth (2023) conceptually replicated the original study by building encoding models that approximate every voxel’s response to a naturalistic two-word phrase as a nonlinear function of the words in the phrase. For each (overlapping) two-word phrase in the natural language stimuli, features were first extracted from a powerful language model, the generative pretrained transformer (GPT; Radford et al., 2018). Then, voxelwise encoding models were trained to learn a linear function from the phrase features to the elicited response after the second word. Using the encoding models, each voxel’s response to a large corpus of over 147,000 two-word phrases was predicted and ranked. This stimulus set comprised both adjective–noun phrases like “red boat” and noun–noun phrases like “picnic table.” Next, for a given phrase selected by a voxel, the first word was replaced with a non-word (i.e., the word was ablated) and the ablated phrase feature was extracted from GPT. Using the learned encoding model, the voxel’s response to the ablated phrase was predicted. Finally, the sensitivity of the voxel to the presence of the ablated word was measured. If the ablated word is important for the voxel to process the phrase, removing it should notably change its response and give high sensitivity. This was done to simulate the compositional versus non-word condition in the original study.

**Figure 3.**
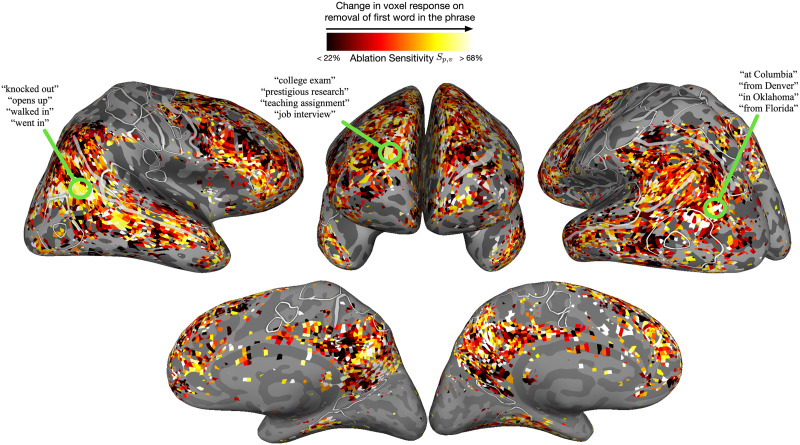
In silico adaptation of a study examining compositional processing in two-word phrases. The original study compared the MEG responses of participants with three different types of two-word phrases: adjective–noun, noun–noun and non-word–noun (Bemis & Pylkkänen, 2011). The in silico simulation of the first two conditions was done by constructing a larger diverse corpus of phrases and using LM-based encoding models to predict fMRI voxel responses to each phrase. The non-word–noun condition was simulated by replacing the first word in a phrase with a non-word (i.e., word ablation), extracting new phrase features from the LM, and then predicting the voxel’s response to the ablated phrase. A large change in a voxel’s response upon word ablation indicated its sensitivity to the first word in the phrase and suggested that the voxel relied on the first word to process the phrase. Similar to the original study, the in silico experiment revealed high sensitivity in ventral medial prefrontal cortex and left anterior temporal lobe. However, the experiment also found that several other areas across cortex combined meaning over the two words in a phrase and moreover, captured diverse semantic concepts arising from the composition. fMRI = functional magnetic resonance imaging; LM = language model; MEG = magnetoencephalography.

The resultant ablation sensitivity of voxels across cortex is visualized in Figure 3. Overall, the in silico experimentation produced similar results to the original study in vmPFC and left anterior temporal lobe—both of these regions exhibit sensitivity to the presence of a compositional word. Beyond areas reported originally, other regions like right inferior parietal and dorsal prefrontal also showed high sensitivity. This finding corroborates other studies of phrase composition (e.g., Boylan et al., 2015; Graves et al., 2010). The in silico study was able to analyze two-word composition in broader regions of cortex by simulating activity for each voxel independently and over a much larger stimulus set that comprises diverse concepts and constructions. While the simulation does not guarantee causal involvement of any region in two-word composition, it demonstrates the utility of broadly sampling stimuli and raises the possibility that many more regions are involved in this process. Moreover, in the in silico study Jain and Huth (2023), this paradigm was extended to much longer phrases (10 words) to understand the relationship between semantic representations and word-level integration across cortex. This would be difficult to implement in real-world settings as doing single-word ablations on increasingly longer phrases is combinatorially explosive.

#### Construction timescale contrast: Vo et al. (2023)

In the third in silico experiment, Vo et al. (2023) tested whether voxelwise encoding models based on features from a neural LM can capture the timescale hierarchy observed during human natural language comprehension. In Lerner et al. (2011), subjects listened to a first-person narrative story that was either intact, reversed, or temporally scrambled at the word level, sentence level, or paragraph level. The scrambling manipulations altered the temporal coherence of the natural language stimulus, and allowed the researchers to measure the reliability of fMRI responses to each condition using intersubject correlation. This revealed an apparently hierarchical organization of temporal receptive windows, with information over short timescales processed in auditory cortex and long timescales processed in parietal and frontal cortex. For the in silico adaptation, the authors trained a multi-timescale long short-term memory (MT-LSTM) network as a language model (Mahto et al., 2020). Then they used the features from the MT-LSTM to predict fMRI responses for each voxel using the data set described above. To mimic the manipulations of the original study, they generated 100 scrambled versions of a held-out test story. This enabled the authors to examine the predicted fMRI responses within each voxel in each subject. Rather than measuring intersubject reliability, they chose to measure an analogous intrasubject reliability value, testing whether the scrambling condition caused a significant drop in this value across conditions. The authors show through simulations that their metric (based on the variance in the simulated fMRI response) is directly analogous to intersubject correlation measures, which is supported by other work (Blank & Fedorenko, 2017; Hasson et al., 2009).

**Figure 4.**
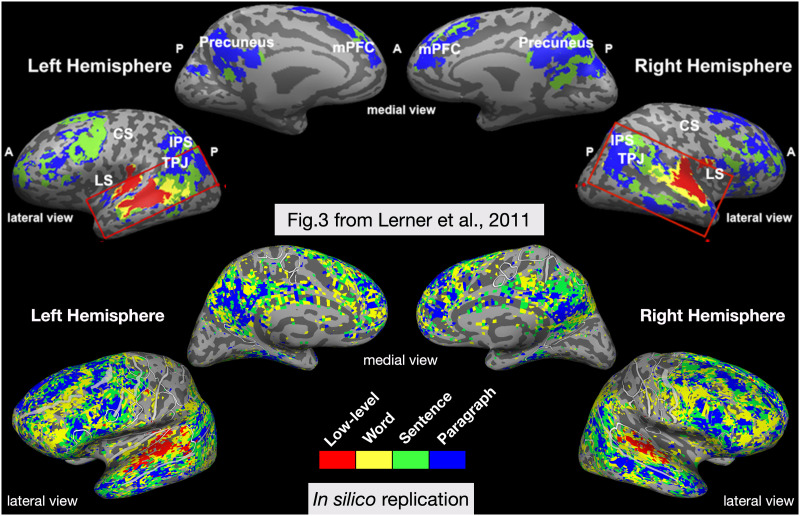
In silico adaptation of a study mapping the hierarchy of temporal receptive windows. (Top) Original results adapted from Lerner et al. (2011). The authors played an audio clip of a narrative story, either intact, reversed, or scrambled at different temporal scales. The figure shows an overlay of several intersubject correlation maps, which measured the cross-subject reliability of the fMRI response in each condition. (Bottom) Results from the in silico experiment of temporal receptive windows, shown for every significantly predicted voxel on a single subject. The in silico experiment suggests that temporal processing windows for different brain regions are not as uniform as previously reported. CS = central sulcus; IPS = intrapariental sulcus; LS = lateral sulcus; mPFC = medial prefrontal cortex; TPJ = temporoparietal junction.

The results of this experiment compared to a schematized version of the original results are shown in Figure 4. This in silico experiment reproduced the pattern of the temporal hierarchy along the temporoparietal axis. It did find that some regions in frontal cortex appear to integrate over shorter timescales than the original work, similar to a later replication of the work (Blank & Fedorenko, 2020) and to a different in-silico replication of the experiment that used GPT-2, rather than a MT-LSTM language model (Caucheteux et al., 2021). Furthermore, the fine-grained resolution of the single-voxel analyses revealed substantial variability across subjects. Taken together, the in silico results suggest that timescales are not as uniform across broad regions as previously reported. This is in agreement with single-neuron studies that show a heterogeneity of intrinsic timescales within a brain region (Cavanagh et al., 2020).

#### Forgetting timescale contrast: Vo et al. (2023)

**Figure 5.**
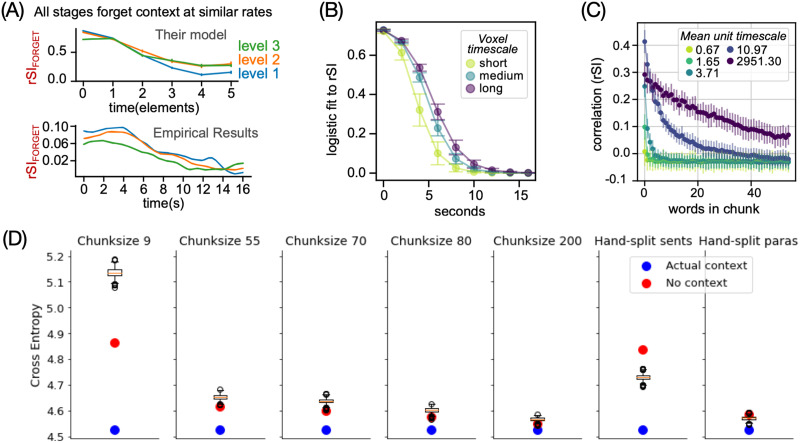
In silico adaptation of a study on forgetting behavior during natural language comprehension. In the original study, Chien and Honey (2020) scrambled paragraphs in a story and analyzed how quickly different brain regions forgot the incorrect context preceding each paragraph. The in silico adaptation used the MT-LSTM based encoding model to predict brain activity at different points in a paragraph when it was preceded by incorrect context. (A) The original study reported that each brain region (denoted by different colored lines) forgot information at a similar rate, despite differences in construction timescales. (B) In contrast, the in silico replication estimated that regions with longer construction timescales also forgot information slowly. (C) Within the MT-LSTM itself, the forgetting rate of different units was related to its attributed timescale. (D) Next, the MT-LSTM’s language modeling abilities were tested on shuffled sentences or paragraphs. The DL model achieved better performance at next-word prediction by using the incoherent, shuffled context as opposed to no context at all. This shows that the DL model retains the incoherent information, possibly because it helps with the original language modeling task it was trained on or because the model has no explicit mechanism to flush-out information when context changes (at sentence/paragraph boundary). The computational model’s forgetting behavior thus differs from the brain, revealing specific flaws in the in silico model that could be improved in future versions, such as a modified MT-LSTM. DL = deep learning; MT-LSTM = multi-timescale long short-term memory; rSI = correlation between scrambled and intact chunks.

In the last experiment, the authors used the same MT-LSTM encoding models as experiment 3 to simulate how different brain regions forget information in naturalistic narrative stimuli (Chien & Honey, 2020). While Chien and Honey found that all brain regions forget information at the same rate (Figure 5A), the in silico results suggested that low-level regions such as auditory cortex forget information at a faster rate than high-level regions like the precuneus (Figure 5B). To better understand this discrepancy, the authors investigated forgetting behavior in the MT-LSTM itself. The results first indicated that every unit in MT-LSTM forgot information at a specific rate tied to its processing timescale (Figure 5C). The authors further hypothesized that the discrepancy could stem from the MT-LSTM’s inability to forget information, even if the preceding context is noisy/uninformative (Figure 5D). To test this, they measured the language model’s cross entropy (lower is better) for a paragraph in three conditions: preceded by the correct paragraph (*actual context*), preceded by no paragraph (*no context*) and preceded by random paragraphs in the story (box plot of 100 different *incorrect contexts*). The story was scrambled by dividing it into non-overlapping chunks of 9, 55, 70, 80, or 200 words or at the actual sentence and paragraph boundaries (*hand-split*). Overall, smaller differences were observed between the conditions as the scrambled context became longer (increased chunk size) and closer to the intact story. With fixed-size chunks, the model performed better when it had no context than when it had access to incorrect information. In contrast, with actual sentences/paragraphs, the model had better performance with incorrect context than no context at all. In both cases, the type of context influences the model performance suggesting that the model retains information from the past. Second, it retains this context even if it is not useful, as in the fixed-chunk conditions. The model could have simply ignored the wrong context to perform better but it did not (or was unable to). This highlights the language model’s inability to forget information that is then reflected in the encoding model results. The authors hypothesized that with hand-split sentences and paragraphs, the incorrect context still provides relevant information to predict the next word, leading to better performance than no context at all.

## DISCUSSION

### Advantages of the in Silico Experimental Design

In the following sections, we discuss the advantages of using in silico experimental design with DL-based encoding models and its potential impact on language neuroscience.

#### Hypothesis development and testing

Each of the studies above conceptually replicated controlled analyses of different linguistic properties using voxelwise encoding models fit on a single naturalistic fMRI data set. Overall, the studies reproduced several effects reported in the original experiments. Interestingly, however, the in silico experiments also found new effects that had not been explored originally. For example, the first experiment suggested that regions in inferior frontal gyrus were more active for concrete words than abstract words. In the second experiment, the investigation of composition in phrases was expanded to a much larger stimulus set and longer phrases. This corroborates earlier results, but the in silico paradigm also enables experimenters to explore interactions between the effect of interest (here, linguistic composition) and other important linguistic properties, such as semantic category. The third experiment found more diversity in timescales in regions like prefrontal cortex than previously reported, closely matching more recent studies of timescale distribution across cortex (Blank & Fedorenko, 2020; Caucheteux et al., 2021) and single-neuron studies (Cavanagh et al., 2020). This demonstrates how the in silico experimental design can be used not only to reproduce and test the generalizability of controlled studies, but also to conduct large-scale exploratory and data-driven analyses that can reveal new aspects of brain function.

Beyond these examples, it is possible to test new hypotheses using in silico experiments before collecting data for a controlled experiment. Wehbe et al. (2018) showcase how BOLDpredictions and in silico experimentation can be used to design new experiments. While the in silico results may not precisely match the eventual data collected on a human population, they would reveal areas where the underlying DL model has failed to match human-level processing and present possible areas of improvement. This has the potential to advance our understanding of both neural network language models and biological language processing. In particular, the in silico paradigm can both draw upon large-scale multidisciplinary efforts to build tools and methods for interpreting neural network language models (Ettinger et al., 2018; Hewitt & Manning, 2019; Ravfogel et al., 2020; Sundararajan et al., 2017), as well as contribute to them by providing a human neural benchmark. Furthermore, more interpretable models allow for novel causal intervention experiments that perturb and control ANNs in ways that biological neural networks cannot be perturbed (Zhang et al., 2022).

#### Testing for generalizability of effects and experimental construct validity

One way to ensure the observed effects of the in silico experiments are not due to the specific task design is to test the generalizability of effects across model architectures, training tasks, neuroimaging data sets and modalities. Unlike reproducibility tests in traditional neuroimaging experiments, these tests do not rely on laborious and time-consuming data collection. Moreover, there are increasingly more tools and techniques to interpret DL models (Clark et al., 2019; Ettinger, 2020), and we can target investigations to these. For example, in the forgetting experiment, the authors checked how the model represented the cognitive process itself. We note that some drawbacks of DL models persist across architectures and tasks. For instance, current language models still perform poorly on common sense reasoning and struggle with capturing long-term dependencies in language. However, with technological advancements, the types of inferences we can make with the in silico paradigm will greatly improve. A case in point is the modeling of contextual processing in the brain. Until recently, language encoding models were largely restricted to static word embeddings that made it difficult to analyze how the brain processed word sequences. However, with the advent of neural language models, this has changed dramatically.

In vision neuroscience, the functional profile of the fusiform face area was established through contrast experiments that evolved over a long period of time (Anzellotti et al., 2014; Gauthier et al., 2000; Kanwisher et al., 1997; Liu et al., 2010; Sergent et al., 1992; Suzanne Scherf et al., 2008; Xu, 2005). Each new experiment was designed to address a confound that was not accounted for previously. Today, however, in silico experiments with vision models have enabled neuroscientists to efficiently contrast large, diverse sets of stimuli and establish the functional specificity of different regions (Ratan Murty et al., 2021). Similarly, in language neuroscience, encoding models have been used to evaluate the semantic selectivity of many regions going beyond semantic contrasts that are tested for a handful of conditions at a time (Huth et al., 2016; Mitchell et al., 2008). This demonstrates how the in silico paradigm allows scientists to quickly design and test multiple experiments that get at the same underlying question. This means that in silico experiments, despite using similar manipulations to controlled experiments, can provide an additional way to test the *construct validity* of the experiment (Yarkoni, 2022). When coupled with generalizability testing, we run a lower risk of over-claiming or over-generalizing.

#### Establishing the validity of model constructs

The in silico approach uniquely facilitates experimenters to evaluate and improve the design of computational models based on observed in silico behavior, going beyond good prediction performance. For example, in the forgetting experiment, the authors identified that the MT-LSTM language model does not instantaneously switch context, and this could influence the observed effects. One possible solution to the nonreplication would be to then train the language model on a new task that encourages forgetting. Alternatively, it could prompt the need for designing alternate architectures that have a built-in forgetting mechanism closer to observed human behavior. Artificial neural networks can be investigated through causal intervention experiments and perturbations, whereas it is very difficult to impossible to do this for human language systems. By analyzing the behavior of DL models in many in silico experiments, we can create a check-and-correct cycle to build better computational models of the brain and establish the validity of model constructs.

An analogous paradigm has also risen in popularity in NLP. Moving beyond better performance with larger language models, there has been a growing effort toward curating diverse language tasks like syntactic reasoning and multistep inference to understand the limitations of current models and establish a benchmark for future innovation. In the same vein, we believe that many different in silico experiments can be used together to establish the validity of different model constructs and provide a benchmark to test future innovations in computational language modeling. We hope that this pushes the field past solely testing encoding model performance on different architectures. Ultimately, this paradigm is a bridge between computational models and experimental designs in neuroscience, such that we can make joint inferences on both and improve them in tandem.

#### Preserving individual participant differences

One potential advantage of in silico encoding model experiments is that the models are typically estimated with single-subject data, allowing experiments to test for effects in individuals rather than over a group average. While group averaging is a common method to improve the signal-to noise ratio (SNR) of neuroimaging studies, it can lead to an underestimation of effect size (Fedorenko, 2021; Handwerker et al., 2004) and hide effects that can be seen with more fine-grained functional anatomical data (Popham et al., 2021). Finally, individual participant analysis does not preclude the estimation of how prevalent an effect is at the population level (Ince et al., 2021); however, it does enable experimenters to account for individual differences, which can be critical to establish links between brain and behavior (Hedge et al., 2018). Consequently, there has been a rising trend toward language studies that analyze participants individually and report consistency of effects across the group (Blank & Fedorenko, 2020; Huth et al., 2016; Wehbe, Vaswani, et al., 2014). While this requires the experimenter to collect more samples per subject to improve the SNR, this approach does not make assumptions about anatomical alignment and preserves spatial resolution important for inferring brain function. The improved sensitivity provides better control for Type 1 errors (by allowing the experimenter to see which effects replicate across participants) and Type 2 errors (by allowing a flexible mapping that can identify important regions in each participant, even if they do not match perfectly in anatomy).

However, the individual-participant analytic approach raises important questions about how to isolate functionally consistent regions across participants and infer consistency of effects. One solution is to use a common set of functional localizer stimuli across participants to isolate functionally homologous networks. For example, the auditory and visual language localizers developed by Fedorenko et al. (2010) and T. L. Scott et al. (2017) have been shown to robustly identify regions across cortex that are important for language processing. This approach enables future studies to consistently isolate language processing regions and characterize their function. Modeling approaches such as hyperalignment (Haxby et al., 2020) and probabilistic mapping of the cortical surface (Huth et al., 2015) offer solutions to compute group-level maps from functional data of individual participants. Nevertheless, these approaches do not provide a computational framework to model individual-participant effects. Encoding models, on the other hand, learn a different function for each brain element in each subject. This enables them to effectively model individual participants and retain high spatial resolution.

#### Improving reproducibility in language neuroscience

There has been an increasing concern in the sciences about the lack of reproducibility for many types of experiments (Pashler & Harris, 2012; Simmons et al., 2011), a problem to which neuroscience is not immune. Several papers have discussed the prevalence of analysis variability, software errors, nontransparent reporting of methods, and lack of data/code sharing as primary causes for low reproducibility and generalizability in neuroscience (see Barch & Yarkoni, 2013, and Poldrack et al., 2020, for introductions to special issues on reproducibility in neuroimaging; Button et al., 2013; Evans, 2017). These studies have also identified issues in statistical analyses, like low statistical power (and, consequently, inflated effect sizes), HARKing, and p-hacking. We believe that the in silico experimentation paradigm can help alleviate some of these issues by providing access to and encouraging open tools for scientific research. When combined with open access to naturalistic data, preprocessing methods, and analysis code, the in silico paradigm can enable scientists to use a standard setup as they test a variety of different hypotheses and thus reduce the “researcher degrees of freedom.” Platforms such as BOLDpredictions can help with this. Indeed, BOLDpredictions is intended as a community tool to allow easy in silico experimentation and generalization testing. It is intended to allow other researchers to contribute their encoding models for other experiments (even outside of language) so that in silico experiments can be available to all. Furthermore, competitions such as Brain-Score (https://www.brain-score.org/competition/) and the SANS’22 Naturalistic fMRI data analysis challenge (https://compsan.org/sans_data_competition/content/intro.html) can align scientific work toward a common goal and facilitate verifiability. Since naturalistic experiments broadly sample the stimulus space, the in silico paradigm can also act as a test bed for generalizability.

### Caveats and the Possibility of Overinterpretation

The in silico paradigm leverages the advantages of both controlled and naturalistic experimental designs with DL-based encoding models. However, it is important to recognize the caveats of this approach so as to minimize the risk of overinterpretation. Here we discuss a number of potential issues.

#### Limitations in the natural language stimulus

One critical advantage of naturalistic stimuli over controlled designs is the access to many diverse examples of language use. However, this also means that the experimenter has little control over the rate of occurrence of different types of linguistic features. Word frequency is an example of uncontrolled variation in natural stimuli (e.g., high frequency of words describe everyday objects like “table” and “book” as opposed to low-frequency words like “artillery” and “democracy”). This presents an important challenge in naturalistic paradigms as the rare variables will have low power and could lead to incorrect or incomplete inferences of brain function. For example, if a voxel encodes semantic concepts related to politics and governance, but this category is not well represented in the naturalistic stimuli, the experimenter runs the risk of incorrectly inferring the voxel function. This can be addressed by building larger, freely available data sets collected from diverse sources and encouraging replication of effects on them.

Another issue with naturalistic paradigms is that they currently rely on the passive perception of language. Many studies have shown that turn-taking in conversation is an important, universal aspect of communication and has implications on how we learn, understand and generate language (Levinson, 2016). Despite a rising trend toward stimuli that take into account social and contextual information, we are still far from studying truly natural use of language with neuroimaging. Some work has investigated aspects of conversational communication (Bögels et al., 2015; Gisladottir et al., 2015; Magyari et al., 2014; Sievers et al., 2020), but the field is still behind in modeling these effects with encoding models or ANNs. Richer data sets will be key to developing these approaches, such as the real-world communication data collected in Bevilacqua et al. (2019) or multimodal movie stimuli discussed in Redcay and Moraczewski (2020). This is an important future direction for the naturalistic paradigm to understand the brain mechanisms of language processing in ethological settings.

#### Limitations in the DL-based feature space

Perhaps the most important factor guiding the in silico experimental design is the success of DL models at predicting brain activity. This paradigm allows neuroscientists to inspect brain function by conducting simulations on the computational model instead, which is easier to perturb, interpret, and control. However, this also means that the types of effects we can observe are limited by the capabilities of the DL model. For example, the forgetting experiment by Vo et al. (2023) demonstrates how the computational model has different behavior than the human brain, affecting the observed in silico behavior. Domain shift presents another common issue for neural networks, although recent studies has proposed that fine-tuning on the target domain/task (Radford et al., 2018) and dynamic data selection during training (Aharoni & Goldberg, 2020; van der Wees et al., 2017) can greatly alleviate this problem for language models. Several encoding model studies explicitly fine-tuned the language model to operate in set (Jain et al., 2020) or trained the language model on a corpus specifically curated to resemble the experimental task (Jain & Huth, 2018; Wehbe, Vaswani, et al., 2014). Furthermore, while ANNs like language models have been successfully employed for a wide range of tasks, their syntactic, common sense, and logical reasoning abilities are still far from those of humans (Ettinger, 2020; Linzen, 2020; Pandia & Ettinger, 2021; Wilcox et al., 2021). Overall, it is important to note that building good encoding models of brain activity and understanding brain function with the in silico paradigm are both contingent on better artificial models of language processing.

#### Limitations in computational modeling

Another source of confounds in encoding models and the in silico paradigm is incorrect modeling assumptions. For example, Jain et al. (2020) highlight that many fMRI encoding models rely on a downsampling technique that incorrectly transforms slowly varying features, making them highly correlated with local word rate. Consequently, an experimenter may (incorrectly) conclude that a brain region that is well predicted by the downsampled features is selective for the slowly varying information (e.g., discourse) it captures, when, in fact, the brain region merely responds to the rate of words. In other cases, it may be important to model several different sources of noise, which has been pursued in other work simulating fMRI data (Ellis et al., 2020). Current neuroimaging modalities also have low SNR, limiting the predictive performance of computational models. Because all modeling approaches likely have caveats and edge cases for which their assumptions fail, it is important to clearly articulate and discuss these issues in future work.

#### Inappropriate causality and zone generalization

Unlike contrast-based experiments and encoding models with simple interpretable features like indicator variables, DL-based encoding models rely on ANNs that are themselves hard to interpret. To this end, any significant correlation observed between brain activity and model predictions leaves many possibilities for interpretation. An experimenter may conclude that the task or objective function the DL model was trained on closely resembles a task the brain solves, when this may not be the case. For example, one might falsely infer that the brain does predictive coding for language because it is well predicted by features from a language model that is trained to predict the next word in a sequence. Guest and Martin (2023) elaborate on this issue by discussing the logical fallacies in inferences drawn between brain behavior or activity, and DL models of language. Specifically, they highlight that studies analyzing parallels between the brain and computational models of language often attribute inappropriate causality by assuming that predictive ability is sufficient to claim task similarity or model equivalence. On the contrary, the direction of causality should be that if an artificial model closely resembles the brain, it can mimic brain behavior and activity, or that a lack of prediction abilities clearly indicates a lack of model equivalence. This is a pertinent issue for in silico experimentation as the paradigm uses computational models of language processing in the brain to simulate its behavior. However, it is important to note that in all of the in silico examples presented here, the authors were using the generalizability of the encoding models to predict brain responses in different conditions. This only suggests that the encoding models can effectively capture the brain’s behavior for language tasks but is not a sufficient account to conclude model equivalence.

Another issue with logical inference in DL-based encoding models relates to the functional equivalence of two brain regions that are both well predicted by a given feature space. In their recent study, Toneva et al. (2022) discuss this issue in detail for language encoding models and provide a computational framework to analyze the extent to which brain regions share computational mechanisms solely based on their encoding performance.

The three main sources of confounds—naturalistic stimuli, DL-based feature spaces, and modeling assumptions—can intersect in interesting ways and raise the probability of incorrect interpretation. False causality stemming from spurious correlations are a problem for in silico experiments, much like controlled experiments. To this end, it is important to emphasize transparent analysis methods, better interpretability tools for DL models, and rigorous tests of reproducibility with diverse data sources.

Although reproducibility is traditionally viewed as the replication of effects across participant pools/data sets, with in silico experimentation we can add another layer of replicability, across different models that have different intrinsic biases (architectures, training objectives, etc.), and learn different types of representation.

### Important Factors of Consideration

Before doing in silico experimentation, one important consideration is determining if the encoding model is “good enough.” While there is no quantitative threshold above which a model can be considered suitable, we suggest the following.

#### Statistical significance of encoding model performance on a diverse, held-out test set

It is imperative that experimenters test whether encoding model performance is statistically significant at the individual brain element level. Any in silico experimentation should only be done on brain elements that are significantly predicted by the computational model. A well-established approach in language encoding modes is to correlate the true and predicted responses for a held-out test set. Following this, a permutation test can be done to check if the correlation is statistically significant.

We also emphasize the importance of using diverse test sets to effectively gauge generalization. If a brain element is selective for features that are not present in the test set, then it may falsely be labeled as poorly predicted. One feasible solution is to use a leave-one-out testing procedure. This can be done by fitting an ensemble of encoding models, each of which excludes one unique set of training data in the model estimation. Statistical significance can then be measured for encoding model predictions on all held-out data. This procedure increases diversity in the test set and improves statistical power.

#### Feature-space selection

Given the diversity of function across the brain, it is possible that no one feature space or computational model best predicts all brain regions. Thus, experimenters should test several different features spaces and models (Nunez-Elizalde et al., 2019) and individually choose the one with best held-out set performance for each brain element. This is especially important for DL models as different neural language models or their layers predict different brain regions well. In this case, we would use the neural language model (layer) that best predicts held-out stories for each element and, further, passes construct validity tests (ie., has well-understood behavior to the controlled manipulation). For example, in the in silico semantic composition experiment, Jain and Huth (2023) found that the lower layers of the neural language model were generally indifferent to ablating words farther in the past (Khandelwal et al., 2018). Consequently, these layers cannot be used to conduct the ablation study, as they do not respond to the manipulation in the first place.

#### Interpreting the DL-models

To establish the validity of computational model constructs, we suggested the use of interpretability tools and techniques to understand how the DL-model itself represents a cognitive process. This would allow the experimenter to directly investigate sources of confounds.

It is also important to consider the types of questions the in silico paradigm is most suited to answer. As demonstrated here, this paradigm can be used to estimate functional properties in the brain, such as selectivity to different word categories or the processing timescale. It cannot, however, be used to test the causal involvement of a brain area or the exact computational mechanism. For example, many regions in the experiments above are shown to capture semantic properties in language. Whether these regions play a causal role in semantic tasks, can only be determined by an in vivo measurement.

### Conclusion and Future Directions

In this article, we highlight the promises of in silico experimentation and detail how it brings together the advantages of controlled experiments and naturalistic experiments paired with encoding models. We showcase four different in silico experiments that all rely on naturalistic language experiments to simulate four different previous studies. We survey the advantages and potential caveats of in silico experimentation and highlight how it can take advantage of recent work in DL to simulate experiments with diverse types of language stimuli.

Current work on DL-based encoding models for language is largely restricted to self-supervised models. This is expected since self-supervised models have been trained on large amounts of data and consequently learn highly useful and transferable linguistic representations. However, it remains to be seen if task-based experimental designs in neuroscience can be simulated and adapted with more goal-directed artificial language networks. Additionally, it is also important to investigate and characterize which types of neuroscientific results can be explored with self-supervised models and what aspects of language meaning are beyond the scope of the next-word-prediction objective.

Lastly, DL-based language encoding models rely on feature extraction from language or speech ANNs (linearizing transform) and learn a linear function atop the features. We believe that the in silico paradigm can become more powerful if language encoding models directly update the parameters of the ANN itself, resulting in an end-to-end system. While this has been popularized in vision (e.g., Bashivan et al., 2019), it is yet to be explored for language. This approach can potentially introduce diversity into the computational mechanisms of the ANNs, such as recurrence, linear readout from a memory store, and so forth, to integrate processing in different brain structures (hippocampus, cortex, etc.). This could allow us to understand parallel mechanisms like linguistic function, working memory access, and attention using this same approach.

## ACKNOWLEDGMENTS

Research reported in this article was also supported by the National Institute on Deafness and Other Communication Disorders of the National Institutes of Health as part of the Collaborative Research in Computational Neuroscience (CRCNS) program. The content is solely the responsibility of the authors and does not necessarily represent the official views of the National Institutes of Health. We thank Nicole Beckage and Javier Turek for useful discussions on this work.

## FUNDING INFORMATION

Shailee Jain, Foundations of Language Fellowship, William Orr Dingwall Foundation. Alexander G. Huth, Burroughs Wellcome Fund (https://dx.doi.org/10.13039/100000861). Alexander G. Huth, Intel Corporation (https://dx.doi.org/10.13039/100002418). Alexander G. Huth, National Institute on Deafness and Other Communication Disorders (https://dx.doi.org/10.13039/100000055), Award ID: R01DC020088. Leila Wehbe, National Institute on Deafness and Other Communication Disorders (https://dx.doi.org/10.13039/100000055), Award ID: R01DC020088.

## AUTHOR CONTRIBUTIONS

**Shailee Jain**: Conceptualization: Lead; Writing – original draft: Lead; Writing – review & editing: Lead. **Vy A. Vo**: Conceptualization: Supporting; Writing – original draft: Supporting; Writing – review & editing: Supporting. **Leila Wehbe**: Conceptualization: Supporting; Funding acquisition: Equal; Supervision: Supporting; Writing – original draft: Supporting; Writing – review & editing: Supporting. **Alexander G. Huth**: Conceptualization: Supporting; Funding acquisition: Equal; Supervision: Lead; Writing – original draft: Supporting; Writing – review & editing: Supporting.

## TECHNICAL TERMS

In silico experimentation: Simulating experiments by predicting brain responses with a computational model to test generalizability and do efficient hypothesis testing.
Naturalistic stimuli: Stimuli that subjects could be exposed to in real life; not artificially constructed for an experiment.
Ecological validity: Determiniation whether an experiment is likely to faithfully reflect and generalize to situations encountered in real life.
Linearized encoding model: Model that learns to predict elicited response in a brain element as a linear function of features of interest extracted from stimuli.
Neural language models: Types of artificial neural networks that learn to predict the next word in a sequence from past context.
Generalizability testing: Testing to see if effects observed on a particular data set extend to a new data set not used for model estimation.
Construct validity: Determination whether a theoretical, experimental, or computational construct faithfully reflects the true phenomena.
Fine-tuning: A secondary learning procedure that modifies an already trained artificial neural network to adapt to a new task or data set.
Zone generalization: Determination whether two brain regions process stimuli similarly.
